# Ankylostomiasis Duodenale

**Published:** 1903-04

**Authors:** A. B. McMillan

**Affiliations:** Burnt Corn, Ala.


### ANKYLOSTOMIASIS DUODENALE.

BY A. B. McMILLAN, M.D.,

BURNT CORN, ALA.

This is an enteric disease, characterized by pallor of skin, malaise, loss of flesh, progressive anemia and the presence of the ankylostoma, a small worm, as above indicated, in the intestinal canal, the female being about two thirds of an inch long and the male varying from one fourth to one half inch in length, both being very small in size. As soon as I began the study of these patients I could not but doubt that few of us know much of the real pathology of this disease. The first that was reported of this disease over our southern country seems to have been by Dr. H. F. Harris of Atlanta, a short notice in *American Medicine*, in July, 1902.

This disease, I have no doubt, has prevailed over southern districts for many years. It is perceptibly more prevalent in some families than others. The season of the year seems to have some influence over this disease, being worse in winter and early spring, and particularly the latter.

Locality, observedly, seems to play some part as a factor. It is not as common among colored as white race. It also seems to occur in families that are ill-fed and poorly-clad.

It is very probable that water is a medium through which it may be transmitted. Dirt-, lime- or chalk-eating is a habit of which many patients give history. Age at which you most frequently discover this condition is from three to fifteen years, and yet it may and does occur at a much more advanced age.

*Symptoms.*—Patients are usually small for their agė. The disease commences with loss of energy, malaise, appetite usually good, digestion often imperfect, patient complaining of some gastric disorder, very slight intestinal disorder, but the latter is not invariable; in some cases obstinate constipation is noticed. The skin is pale, and later becomes more of a bluish tint. The conjunctivæ demonstrate that the blood is scanty and very much paler than normal. The tongue is flat, beefy and exceedingly pale.

Bronchial irritation is sometimes present. Shortness of breath is an early symptom.

Patient loses flesh, and as the disease progresses more pronounced anemia ensues.

At first there are rarely any cardiac symptoms, but later palpitation and some pain in precordial region. Acceleration of the heart's action is noted, the pulse becoming soft, rapid and weak. Night sweats also occur, and occasionally disturbances of temperature. Nervous symptoms are not well-defined; patient complains of headache. Not infrequently patient has neuralgia coming on in almost intolerable paroxysms.

During the progress of this disease the above symptoms become pronounced and aggravated. Edema of the extremities, face and around the eyelids is noticed. The skin grows more waxy in appearance, and the patient becomes more alarmed at his perilous condition, usually lying in a somewhat stupid manner and muttering as though suffering great agony, and yet I doubt whether very much pain is present. Rarely there occur periods of restlessness and even excitement. You also not infrequently notice some tenderness over umbilical region. Tympanites is at times present ; patient rapidly growing weaker and dying from exhaustion.

Any or all of the above symptoms may be noticed in a case in which the ankylostoma is present.

Abscess of the liver is a not infrequent complication.

Diagnosis can be made from the chains of symptoms above named and the examination by the microscope.

Differential diagnosis must be made from idiopathic anemia, leucocythemia, nephritis, organic lesions of the heart, tuberculosis and tubercular peritonitis.

Duration of this disease varies. Patient may succumb in a few months or may live for a number of years.

Treatment consists in correcting habits of patients, if any are found. The patient is starved by the enormous nnmber of the little worms which are present and almost prevent the assimilation of any nourishment, and which are gradually sapping the life of the patient.

We see no reason why our treatment in future may not yet be accompanied by brilliant results, and that we may be relieved of much embarrassment in care of such patients. Treatment now generally accepted is largely antiseptic and about as follows:

On day before your antiseptic is given, the patient eats only a small dinner, and in the afternoon he is given a good course of calomel and is allowed no supper. If the bowels move well in the night, as soon as patient awakens in the morning you administer powdered thymol gr. xxx, and in two hours again give grs. xxx of this drug. The patient is allowed no breakfast and no dinner, and must remain in bed all day, and late in the evening, about 5 or 6 o'clock, you give a good dose of salts. If on the night before the antiseptic is given the bowels do not move well, more purgative medicine must be given until this is accomplished.

